# Studies of *Myxidium giardi* Cépède, 1906 infections in Icelandic eels identifies a genetically diverse clade of myxosporeans that represents the *Paramyxidium* n. g. (Myxosporea: Myxidiidae)

**DOI:** 10.1186/s13071-018-3087-y

**Published:** 2018-10-22

**Authors:** Mark A. Freeman, Árni Kristmundsson

**Affiliations:** 1 Ross University School of Veterinary Medicine, Basseterre, Saint Kitts and Nevis; 2 Institute for Experimental Pathology, University of Iceland, Reykjavík, Iceland

**Keywords:** *Paramyxidium* n. g., *Paramyxidium giardi* n. comb., Elopomorpha, Protacanthopterygii, Myxosporea, *Chloromyxum*

## Abstract

**Background:**

The myxosporean *Myxidium giardi* Cépède, 1906 was described infecting the kidney of the European eel, *Anguilla anguilla* (L.), having spindle-shaped myxospores and terminal sub-spherical polar capsules. Since then, numerous anguillid eels globally have been documented to have similar *Myxidium* infections. Many of these have been identified using the morphological features of myxospores or by the location of infection in the host, and some have been subsequently synonymised with *M. giardi*. Therefore, it is not clear whether *M. giardi* is a widely distributed parasite, infecting numerous species of eels, in multiple organs, or whether some infections represent other, morphologically similar but different species of myxosporeans. The aim of the present study was to assess the status of *M. giardi* infections in Icelandic eels, and related fish hosts in Malaysia and to use spore morphology and molecular techniques to evaluate the diversity of myxosporeans present.

**Results:**

The morphologies of the myxospores from Icelandic eels were very similar but the overall dimensions were significantly different from the various tissue locations. Myxospores from the kidney of the Malaysian tarpon, *Megalops cyprinoides* (Broussonet), were noticeably smaller. However, the *SSU* rDNA sequences from the different tissues locations in eels, were all very distinct, with percentage similarities ranging from 92.93% to as low as 89.8%, with the sequence from Malaysia being even more dissimilar. Molecular phylogenies consistently placed these sequences together in a clade that we refer to as the *Paramyxidium* clade that is strongly associated with the *Myxidium* clade (*sensu stricto*). We erect the genus *Paramyxidium* n. g. (Myxidiidae) to accommodate these histozoic taxa, and transfer *Myxidium giardi* as *Paramyxidium giardi* Cépède, 1906 n. comb. as the type-species.

**Conclusions:**

There is not a single species of *Myxidium* (*M. giardi*) causing systemic infections in eels in Iceland. There are three species, confirmed with a robust phylogeny, one of which represents *Paramyxidium giardi* n. comb. Additional species probably exist that infect different tissues in the eel and the site of infection in the host fish is an important diagnostic feature for this group (*Paramyxidium* n. g. clade). Myxospore morphology is generally conserved in the *Paramyxidium* clade, although actual spore dimensions can vary between some species. *Paramyxidium* spp. are currently only known to infect fishes from the Elopomorpha.

## Background

The importance of the shape of myxospores and its relative usefulness in myxosporean taxonomy has been scrutinised in recent years, and it has been unambiguously demonstrated that numerous genera are now polyphyletic in molecular phylogenetic analyses due to the use of spore morphology as the principal taxonomic measure [1–3]. However, myxospores remain the primary diagnostic feature of myxosporean infections in fish and other vertebrates [4] and their morphology, especially when combined with other important information from the host, can sometimes be sufficient to make preliminary identifications or diagnoses without the use of DNA analysis. The polyphyletic nature of some myxosporean genera in molecular analyses has occurred, in part, as our systematic framework for deciding which family and genus to place novel species has become less stringent over time, and gives priority to basic myxospore morphology over other characters [4]. This is combined with the fact that there also seems to be a general reluctance to establish new genera when necessary, with the historical preference being to further loosen the descriptive boundaries of existing genera [4]. This has led to artificially high numbers of species being placed in certain genera, which in some cases are clearly not closely related to each other, and has hence contributed to polyphyly in subsequent molecular phylogenies [2, 4].

*Myxidium giardi* Cépède, 1906 was originally described from the kidney of the European eel, *Anguilla anguilla* (L.), in France, with myxospores described as spindle-shaped with terminal sub-spherical polar capsules [5], but have since been reported in numerous sizes and described as both wide and slender, some with almost spherical polar capsules [6, 7]. Globally, numerous species of anguillid eels have been reported to have similar *Myxidium* spp. infections, many of which have been distinguished using morphological features such as spore size and number or lack of valvular striations [8, 9] or the site of infection in the eel (skin, gills, kidney, stomach, etc.) [10]. However, many descriptions of *Myxidium* spp. infecting eels have subsequently been synonymized with *M. giardi*, as less emphasis was placed on details such as the site of infection [11] and *M. giardi* was considered to have an almost worldwide distribution [12]. Currently, it is not clear whether *M. giardi* is a widely distributed parasite infecting numerous species of eels, in multiple organs, or whether some infections represent other, morphologically similar, species of myxosporeans. One short rDNA sequence exists for *M. giardi* from the urinary system of *A. anguilla* from Scotland, and phylogenetic analyses place this in the freshwater urinary clade, with numerous other myxosporeans found infecting the urinary systems of fish, but none with a similar spore morphology [13].

Anguillid eels (Order Anguilliformes) are catadromous fish consisting of 17 species in a single genus, *Anguilla* Garsault, distributed in all the world’s oceans. Two species inhabit the Atlantic Ocean, the European eel, *A. anguilla*, and the American eel, *Anguilla rostrata* (Lesueur) [14]. A dramatic decline has been experienced in populations of the European eel and diseases/parasites are considered to be one of several factors responsible. Consequently, since 2009, the European eel has been on the red list of the International Union for the Conservation of Nature (IUCN) and hence is listed as a ‘critically endangered’ species [15]. The Indo-Pacific tarpon, *Megalops cyprinoides* (Broussonet) (Order Elopiformes), inhabits tropical coastal and brackish waters of the Indo-Pacific, migrating between the open sea and inland rivers and mangroves [16]. Elopiformes are related to, but do not resemble anguillid eels, and like eels they spawn at sea producing leptocephalic larvae that later migrate inland [17]. Tarpon (Elopiformes) together with their sister group the eels (Anguilliformes) form the Elopomorpha which are one of the oldest major extant teleost lineages [18].

In the present study, we aimed to examine eels from Iceland and genetically characterise *Myxidium giardi-*like parasites from different infection sites in the hosts. In addition, we aim to compare these with morphologically similar parasites previously observed by one of us (MAF) in tarpon from Malaysia.

## Methods

Thirty-one live European eels, *A. anguilla*, (growing yellow eels; total length range 42–68 cm) were collected from Lake Vifilsstadavatn in Iceland during May 2013 using fyke nets. The fish were transported to the laboratory, placed in tanks with freshwater and kept alive until examination. Prior to examination, the fish were euthanized in MS-222. All eels were dissected and examined for myxosporeans in the gills, kidney and stomach wall using dissecting and compound microscopes. Images were taken of twenty spores from each tissue location for size estimations using ImageJ. In addition, fourteen mature Pacific tarpon, *Megalops cyprinoides* total length range 22–34 cm, were caught with gill nets in Kilim mangroves on Langkawi Island, Malaysia. Tarpon were examined for gill and kidney myxosporeans, using dissecting and compound microscopes. In Malaysia, myxospores were photographed in the field using a portable compound microscope and a Dino-Eye eyepiece camera. Infected tissues from eels and tarpon were fixed in 10% buffered formalin and processed for standard histology, 3 μm thick sections prepared, stained with Giemsa and mounted in resin-based medium.

Oligochaetes (*n* = 100) were taken from sediment samples from the lake Vifilsstadavatn in Iceland, isolated and incubated at 10 °C in filtered (pore size 0.45 μm) lake water in 24-well tissue culture plates. Oligochaetes were examined daily using a dissecting microscope for the presence of actinospore production, typically characterized by a cloudy secretion from the anus of the worms, and confirmed using a compound microscope. Every other day, the water in the wells was exchanged. The oligochaetes that produced actinospores were used for DNA analysis and images of the actinospores taken as detailed above.

Infected tissue (kidney, stomach wall) or spore-filled cysts (gills), equal to approximately 20–40 mg, were placed directly into DNA lysis buffer for molecular analysis. Total DNA was extracted using a GeneMATRIX DNA isolation kit (EURx, Gdansk, Poland) following the tissue protocol and used as templates in subsequent PCR reactions. Parasite small subunit ribosomal DNA (*SSU* rDNA) was amplified using the general myxosporean primers and methodology previously described [2] with a new reverse primer M-790r (5'-ACG ACC AAT TAA GGC TAT GC-3'), paired with 18e, utilising the PCR conditions as in [2]. In addition, the primer pair Mg-50f (5'-ACT AAG CCA TGC ATG TCT ATG T-3') and Mg-1170r (5'-TGA TCA ATC GAA ACG GTC TAG G-3') were designed to assist in further studies of these myxosporeans from anguillid eels and amplified a 1150 bp region of the *SSU* rDNA including the phylogenetically informative variable V1-V5 regions; PCR conditions were the same as in [2], except for using an annealing temperature of 58 °C and extension time of 1 min. PCRs were conducted on parasite DNA, from each tissue, from 4 different fish and amplified in triplicate. PCR products of the expected sizes were recovered using a GeneMATRIX PCR products extraction kit (EURx Poland) and sequencing reactions were performed using BigDyeTM Terminator cycle sequencing chemistry utilising the same oligonucleotide primers that were used for the original PCRs. DNA sequencing was performed in both forward and reverse directions for all PCR products and nucleotide BLAST searches performed for each sequence read to confirm a myxosporean origin [19]. The contiguous sequences were obtained manually using CLUSTAL X and BioEdit [20, 21]. CLUSTAL X was used for the initial *SSU* rDNA sequence alignments of the novel sequences to other related myxosporean sequences identified from the BLAST searches and from relevant literature [1, 13, 22, 23]. Percentage divergence matrices were constructed from selected aligned taxa in CLUSTAL X using the neighbour-joining method based on the Kimura 2-parameter model [24].

Phylogenetic analyses were performed using the maximum likelihood methodology in PhyML [25] with the automatic smart model selection (selection criterion: Akaike Information Criterion), running the general time-reversible substitution model (GTR+G6+I) with 1000 bootstrap repeats. Bayesian inference (BI) analysis was performed using MrBayes v. 3.2 [26]. For the BI analysis, models of nucleotide substitution were first evaluated for the alignment using MrModeltest v. 2.2 [27]. The most parameter-rich evolutionary model based on the AIC was the general time-reversible, GTR+I+G model of evolution. Therefore, the settings used for the analysis were nst = 6, with the gamma-distributed rate variation across sites and a proportion of invariable sites (rates = invgamma). The priors on state frequency were left at the default setting (Prset statefreqpr = dirichlet (1,1,1,1)), and *Chloromyxum leydigi* (AY604199) was set as the outgroup. Posterior probability distributions were generated using the Markov Chain Monte Carlo (MCMC) method with four chains being run simultaneously for 1,000,000 generations. ‘Burn-in’ was set at 2500 and trees were sampled every 100 generations making a total of 7500 trees used to compile the majority rule consensus trees.

Measurements of myxospores from each organ (kidney, gills and stomach wall) were tested for normality using the Shapiro-Wilk test. To test whether there was a significant difference in analogous measurement between the different myxospores, a Student’s t-test was applied. Both tests were performed in RStudio (version 0.98.1062).

## Results

All the fish examined in this study appeared in good condition and no gross signs of a disease were present. From the 31 eels examined, small myxosporean cysts were observed on the gills of 25 fish (prevalence of 83%) using a dissection stereoscope, ranging between 2–10 cysts per gill arch. Infections in the kidney and the stomach wall were only detected using a compound microscope. Spores were detected in the kidney of 10 fish (32%) and from stomach scrapings from 6 eels (19%). Infections were light in most cases, being more apparent in the gills. All myxospores detected had a similar morphology that conformed to the currently accepted shape and size range for *Myxidium giardi* (Table 1) [12]. No gill cysts were observed from 14 tarpon, examined in Malaysia, but the kidney of 4 fish (29%) were heavily infected with a myxosporean, observed in fresh tissue preparations. The myxospores from tarpon kidney had a similar morphology to the ones from the eels. However, they were noticeably, and significantly, smaller than all three species observed from the Icelandic eels (Tables 1 and 2).

**Table 1:** Measurements of twenty fresh myxospores of *Paramyxidium* spp., including dimensions from the original description of *P. giardi*

Species	Host species	Site of infection	Spore body Range (Mean)		Polar capsules Range (Mean)		Reference
			Length	Width	Length	Width	
*P. giardi* ^a^	*A. anguilla*	Kidney	9.0–10.0 (nd)	5.5–6.0 (nd)	3.0–5.0 (nd)	nd (2.0)	Cépède (1906) [5]
*P. giardi*	*A. anguilla*	Kidney	9.5–11.4 (10.6)	6.5–7.5 (7.0)	3.5–4.2 (4.0)	2.7–3.9 (3.6)	Present study
*P. magi* n. sp.	*A. anguilla*	Stomach wall	10.8–12.9 (11.6)	7.0–8.4 (7.6)	3.6–4.6 (4.0)	3.1–4.0 (3.6)	Present study
*P. branchialis* n. sp.	*A. anguilla*	Gills	10.7–12.3 (11.6)	6.6–7.8 (7.3)	3.9–4.5 (4.2)	3.2–4.2 (3.8)	Present study
*P. bulani* n. sp.	*M. cyprinoides*	Kidney	6.1–6.9 (6.7)	4.2–5.1 (4.7)	2.1–2.5 (2.3)	1.5–2.0 (1.8)	Present study

^a^Original description

*Abbreviation*: nd, no data

**Table 2:** Statistical comparison of spore length (above the diagonal) and width (below the diagonal) of the four species observed in the study

Species	*P. giardi*	*P. magi* n. sp.	*P. branchialis* n. sp.	*P. bulani* n. sp.
*P. giardi*		*t*_(19)_ = -6.53; *P* < 0.0001 ****	*t*_(19)_ = -6.42; *P* < 0.0001 ****	*t*_(19)_ = 30.64; *P* < 0.0001 ****
*P. magi* n. sp.	*t*_(19)_ = -6.22; *P* < 0.0001 ****		*t*_(19)_ = -0.45; *P* = 0.6544 ns	*t*_(19)_ = 51.94; *P* < 0.0001 ****
*P. branchialis* n. sp.	*t*_(19)_ = -3.61; *P* = 0.0009 ***	*t*_(19)_ = 3.18; *P* = 0.0029 **		*t*_(19)_ = 44.90; *P* < 0.0001 ****
*P. bulani* n. sp.	*t*_(19)_ = 29.38; *P* < 0.0001 ****	*t*_(19)_ = 31.12; *P* < 0.0001 ****	*t*_(19)_ = 33.70; *P* < 0.0001 ****	

*Abbreviation*: ns, not significant

**P < 0.01, ***P < 0.001, ****P < 0.0001

**Fig. 1:**
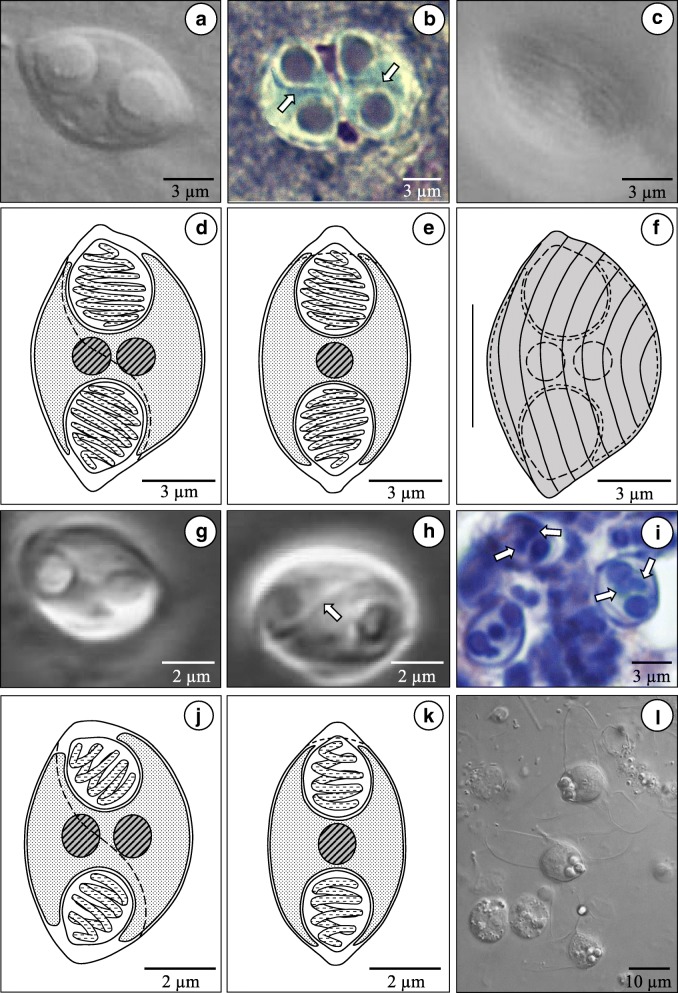
**a**-**c** *Paramyxidium magi* n. sp. from the stomach wall of *Anguilla anguilla*. Fresh spore in valvular view showing the polar capsules enclosing the polar filaments (**a**), stained imprint of two spores, in the sutural plane, showing a sigmoidal sutural line (arrows) (**b**) and a fresh spore with numerous valvular striations (**c**). **d**-**f** Line drawings of *P. giardi* from the kidney of an eel; in sutural view (**d**), valvular view (**e**) and one with the valvular striations (**f**). **g**-**k** *P. bulani* n. sp. from the kidney of *Megalops cyprinoides*. **g**, **h** Fresh spores in sutural view, showing the polar capsules (**g**) and the sutural line (arrow) (**h**). **i** Histological sections showing the two parallel nuclei (arrows) between the polar capsules. **j**, **k** Line drawings in sutural (**j**) and valvular (**k**) views. **l** Fresh Aurantiactionmyxon type actinospores from the oligochaete *Lumbriculus variagatus* from Lake Vifilsstadavatn in Iceland

The overall morphology and dimensions of the myxospores from Icelandic eels appeared similar (Fig. 1). However, taking average dimensions from 20 measured spores, they were significantly different with regard to spore length and/or width (Tables 1 and 2). Most fresh spores were typically observed in the sutural plane and had an oval to bluntly-rounded shape with almost spherical polar capsules opening sub-terminally (Fig. 1a-e). The sutural line, when visible, was sigmoidal and striations were visible on the surface of the spore valves (Fig. 1c, f), although these structures were not always clearly visible. In the valvular view, spores were more lemon-shaped than a true spindle or fusiform shape (Fig. 1e), but clearly resembled the original drawings by Cépède [5], however they did not have terminal capsular foramina. All myxospores in this study were between 62–70% as wide as they were long, which is similar to the original description of *Myxidium giardi* from eels in France (Table 1) [5]. The myxospores from tarpon in Malaysia, had a remarkably similar overall morphology to those from Icelandic eels (Fig. 1g-k), but they were significantly smaller in size (Tables 1 and 2). When myxospores were stained, it was clear that the sporoplasm contained two nuclei, in all cases, as originally described for *M. giardi* [5]. Only one oligochaete, morphologically identified as *Lumbriculus variegatus*, produced actinospores with an Aurantactinomyxon morphology (Fig. 1l, Table 3).

**Table 3:** Measurements of twenty fresh actinospores observed in this study

Actinospore type	Host species	Diameter of spherical spore body Range (Mean)	Length of caudal process Range (Mean)	Width of caudal process Range (Mean)	Largest span between end of caudal processes Range (Mean)	Reference
Aurantiactinomyxon	*L. variegatus*	9.0–12.5 (10.4)	14.5–16.3 (15.4)	7.5–10 (8.5)	29.9–32.5 (30.8)	Present study

### Histological examination

**Fig. 2:**
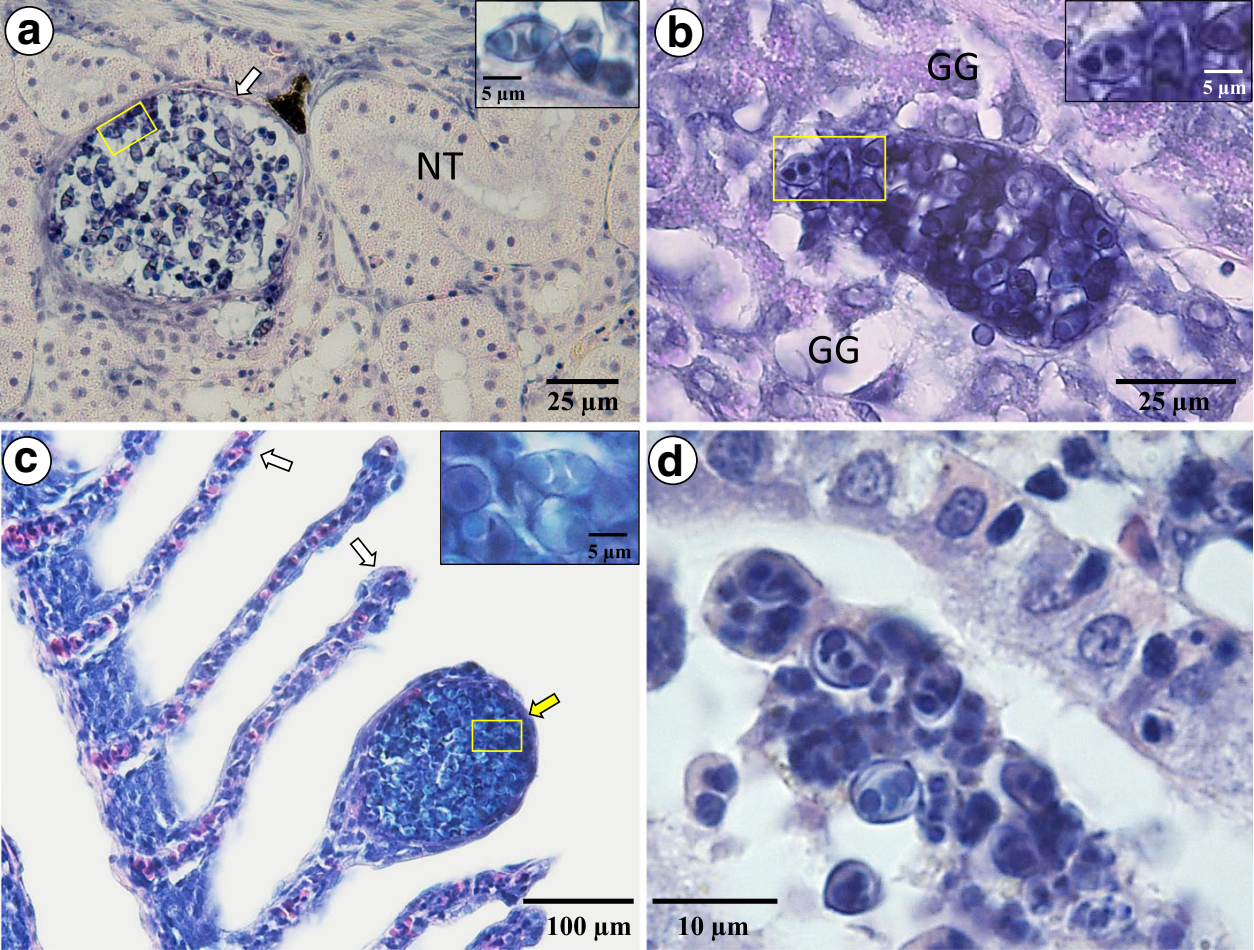
**a** A polysporous plasmodium of *Paramyxidium giardi* inside a kidney tubule of *Anguilla anguilla*, where is causes extensive widening of the tubules causing atrophy and necrosis of the tubular epithelial cell (arrow); insert shows higher magnification of spores from within the yellow box. **b** A polysporous plasmodium of *P. magi* n. sp. in the gastric gland of stomach wall; insert shows higher magnification of spores from within the yellow box. **c** Normal secondary gill lamellae of *A. anguilla* (white arrow) and one infected with *P. branchialis* n. sp. (yellow arrow). The large plasmodium causes disruption of epithelium and the pillar cells of the secondary lamella; insert shows higher magnification of spores from within the yellow box. **d** *P. bulani* n. sp. infection in the kidney of a Pacific tarpon. The parasite develops inside the kidney tubules, often attached to the brush border of the tubular epithelial cells, without causing any apparent pathology. *Abbreviations*: GG, gastric glands; NT, normal tubule

*Paramyxidium giardi* infecting the kidney of eels forms polysporous plasmodia which vary greatly in size (*c.*55–120 μm in this study). The plasmodia were most commonly found as intratubular, both in the proximal and distal kidney tubules, although they were also, to a lesser extent, observed in the renal interstitium. The associated histopathology was mostly due to mechanical disruption of the tissue as the large plasmodia often caused extensive widening of the kidney tubules followed by atrophy and degeneration of the tubular epithelial cells (Fig. 2a) which is in agreement with the pathology described by Copland [28]. Renal infections described in his study [28] were more extensive than in this study. Consequently, he observed varying degrees of pathology in other parts of the kidney, e.g. the bowman capsule and the kidney interstitium.

*Paramyxidium magi* n. sp. is histozoic in the stomach wall. It forms polysporous plasmodia of variable sizes (*c.*60–95 μm) in the gastric gland interstitium (Fig. 2b). Large spore masses in the gastric glands cause focal compaction and disruption of the adjacent gastric glands similar to previous reports [28].

*Parayxidium branchialis* n. sp. is histozoic in the gills. As observed by Copland [28], the great majority of infections were observed in the secondary lamellae. The plasmodia are polysporous and differ significantly in size (*c.*35–142 μm). Infections cause separation of the lamellar epithelial cells from the basement membrane and disruption of pillar cells (Fig. 2c) and in some cases oedema in the basal part of the secondary lamellae.

*Paramyxidum bulani* n. sp. infects the kidney. The plasmodia are polysporous, somewhat elongated (size range: length, 25–75 μm; width, 10–22 μm) and develop as intratubular, both in proximal and distal tubules. They are seen at various stages in development, some of which are fully mature, and commonly attached to the brush border of the proximal kidney tubule epithelium. In the distal kidney tubules, the proportion of mature plasmodia seem higher (Fig 2d). No pathology was associated with infections.

No host response, such as encapsulation by fibroblasts or infiltration of immune cells, was detected in association with any of these four different myxosporeans.

### Molecular analyses

*SSU* rDNA sequences were successfully sequenced for *P. giardi* (2076 bp) and for the three species recognised here as new, i.e. *P. magi* n. sp. (2070 bp), *P. branchialis* n. sp. (2082 bp), *P. bulani* n. sp. (2056 bp) and the Aurantiactinomyxon (865 bp), which have been assigned accession numbers in GenBank: MH414925-MH414929. BLAST searches confirmed a myxosporean origin for all sequences with closest matches in the database being to numerous actinosporean sequences and also *Myxidium lieberkuehni* Bütschli, 1882, the type-species of the genus *Myxidium*. The *SSU* rDNA sequences from infected tissues from eels, were all very distinct, with percentage similarities ranging from 92.93% to as low as 89.8%, over > 2000 bp of sequence data (Table 4). The *SSU* rDNA sequence from *P. bulani* n. sp. was even more dissimilar, being < 84% similar to any of the other myxospore sequences, and only 81.75% similar to the kidney-infecting *P. giardi* from eels (Table 4); only *M. lieberkuehni* was more dissimilar, being < 79% similar to any of the sequences generated from myxospore infections (Table 4). The Aurantiactinomyxon sequence did not match any of the sequences generated from the myxospores in this study and was most similar (96.07%) to *P. magi* n. sp.

**Table 4:** Percentage identities of *SSU* rDNA sequences (above the diagonal) and number of bases compared (below the diagonal) for the new sequences in the *Paramyxidium* clade (bold), related actinospores and the type-species of the genus *Myxidium*, *M. lieberkuehni*

		1	2	3	4	5	6	7	8
1	***Paramyxidium giardi***	–	92.93	89.80	98.29	89.91	94.79	81.75	77.98
2	***Paramyxidium magi*** **n. sp.**	2051	–	91.34	92.50	91.12	96.07	82.61	78.90
3	***Paramyxidium branchialis*** **n. sp.**	2058	2055	–	89.17	95.77	90.35	83.08	78.70
4	*Aurantiactinomyxon* (AF483598)	1931	1933	1930	–	89.58	94.07	81.27	77.60
5	*Synactinomyxon* (AY787784)	1605	1599	1606	1602	–	91.31	82.15	77.53
6	***Aurantiactinomyxon***	864	865	860	860	863	–	83.55	80.05
7	***Paramyxidium bulani*** **n. sp.**	2044	2041	2039	1917	1585	863	–	78.70
8	*Myxidium lieberkuehni* (X76638)	2021	2014	2028	1893	1562	827	2009	–

**Fig. 3:**
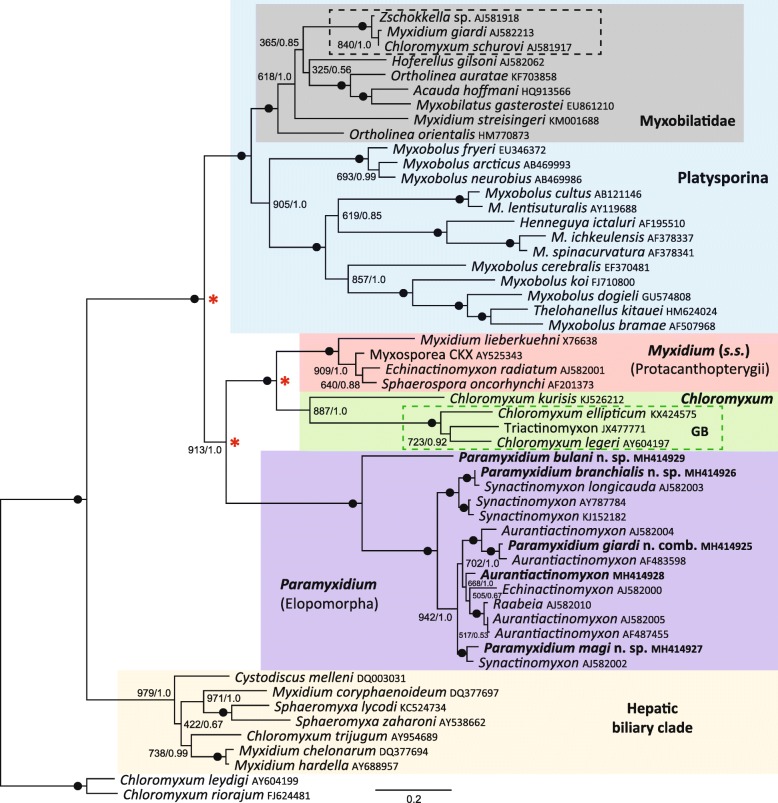
Maximum likelihood topology of small subunit ribosomal DNA from 54 myxosporeans, rooted to *Chloromyxum leydigi* and *C. riorajum* (infecting cartilaginous fishes). Bootstrap support values and posterior probabilities are shown at the nodes; solid black dots indicate full support for that node. Novel sequences from this study are in bold and contained within the *Paramyxidium* Clade (lilac box). The *Paramyxidium* clade forms a robust sister clade relationship with *Myxidium* clade (*sensu stricto*) (red box) and the *Chloromyxum* clade (green box). The dashed green box within the *Chloromyxum* clade shows species that infect the gall-bladder (GB) of cypriniform fishes. Nodes with a red asterisk indicate that the common ancestor was likely to have been a renal-infecting myxosporean. The Platysporina (blue box) forms a fully supported sister clade to this *Paramyxidium* / *Myxidium* (*s.s*.) / *Chloromyxum* grouping and includes the Myxobilatidae (grey box). Highlighted within the Myxobilatidae, by a dashed black box, is the *Chloromyxum schurovi* sub-clade that contains other renal isolates from eels that have been reported as *Myxidium giardi* and *Zschokkella* sp. Basal to these main clades is the hepatic biliary clade, a well-supported clade which contains numerous taxa, some of which infect vertebrates other than fish

Molecular phylogenetic analyses produced trees with very similar topologies irrespective of the methodology used (Fig. 3). All *Paramyxidium* spp. were fully supported in a clade with numerous actinosporean sequences (*Paramyxidium* clade) shown as the lilac box in Fig. 3. *Paramyxidium bulani* n. sp. from a tarpon in Malaysia, represented the basal sequence of the clade. The *Myxidium* clade (*sensu stricto*), that includes the type-species *M. lieberkuehni*, was also fully supported (red box, Fig. 3) and formed a fully supported sister group to the clade containing *Chloromyxum* spp. (green box, Fig. 3). Together these formed a robustly supported sister clade to the *Paramyxidium* clade. The Platysporina (blue box, Fig. 3) formed a fully supported sister clade to this *Paramyxidium* / *Myxidium* (*s.s*.) / *Chloromyxum* grouping. Basal to this is the hepatic biliary clade, a well-supported clade which contains numerous taxa currently assigned to the Myxidiidae, some of which infect vertebrates other than fish [22].

### Taxonomy

**Class Myxosporea Bütschli, 1881**

**Suborder Variisporina Lom & Noble, 1984**

**Family Myxidiidae Thélohan, 1892**

**Genus** ***Paramyxidium*** **n. g.**

### Diagnosis

Myxospore length width ratio between 1.4–1.6:1 resulting in a lemon-shaped form in valvular aspect and oval to bluntly-rounded form in sutural view. Valvular striations present, with sigmoidal sutural line. Polar capsules two, almost spherical. Two nuclei in the sporoplasm positioned between polar capsules. Capsular foramina open sub-terminally. Histozoic polysporous plasmodia develop in various host tissues with different species infecting different host tissues.

***Type-species*:** *Paramyxidium giardi* (Cépède, 1906) n. comb.

***Other species*:** *Paramyxidium branchialis* n. sp.; *Paramyxidium bulani* n. sp.; *Paramyxidium magi* n. sp.

***Etymology*:** The new genus is named *Paramyxidium* n. g. as it is placed next to the type-species of the genus *Myxidium* (*s.s.*) in phylogenetic analyses.

***ZooBank registration***: To comply with the regulations set out in article 8.5 of the amended 2012 version of the *International Code of Zoological Nomenclature* (ICZN) [29], details of the new genus have been submitted to ZooBank. The Life Science Identifier (LSID) of the article is: urn:lsid:zoobank.org:pub:716F81BB-DAFC-4B33-89ES-A57684065765. The LSID for the new genus name *Paramyxidium* is: urn:lsid:zoobank.org:act:27B9FBC6-345A-4DD9-93D6-A13EACB82A18.

### Remarks

*Paramyxidium* spp. are sufficiently different with respect to either myxospore morphology/ characteristics, tissue location, host type or DNA sequences to necessitate the erection of a novel genus within the family Myxidiidae Thélohan, 1892 to accommodate them. The family Myxidiidae currently contains five genera: *Myxidium* Bütschli, 1882; *Coccomyxa* Léger & Hesse, 1907; *Cystodiscus* Lutz, 1889; *Enteromyxum* Palenzuela, Redondo & Alvarez-Pellitero, 2002; *Soricimyxum* Prunescu, Prunescu, Pucek & Lom, 2007; and *Zschokkella* Auerbach, 1910. Species of *Cystodiscus* and *Soricimyxum* are only found in non-fish hosts [22]. *Enteromyxum* spp. have very large and elongated polar capsules, spores that lack valvular striations, and are phylogenetically associated with morphologically similar forms from the gastrointestinal tract of marine fishes [4]. *Coccomyxa* spp. are typically found in the gall-bladder of marine fishes, are more ellipsoidal in morphology, and are phylogenetically distantly placed in the marine gall-bladder (marine *Myxidium*) clade [2]. The genera *Myxidium* and *Zschokkella* are more complicated to decipher for as both are polyphyletic, with many species likely being wrongly attributed to each genus [1, 2, 4]. However, DNA sequence data convincingly placed all *Paramyxidium* taxa in a robust clade which is sister to the *Myxidium* clade (*s.s.*). In addition, host differences and tissue tropism are apparent, with *Paramyxidium* taxa infecting the Elopomorpha in numerous organs and *Myxidium* spp. (*s.s.*) infecting the renal system of members of the Protacanthopterygii.

Therefore, we erect the genus *Paramyxidium* to accommodate these myxosporeans, and transfer *Myxidium giardi* as *Paramyxidium giardi* Cépède, 1906 n. comb. as the type-species. The annelid hosts for *Paramyxidium* spp. are likely to be oligochaetes. Currently known species are restricted to freshwater environments and fish hosts from the Elopomorpha, presumably becoming infected during migratory periods into fresh water.

### *Paramyxidium magi* n. sp.

***Type-host*:** *Anguilla anguilla* (L.) (Anguilliformes: Anguillidae).

***Type-locality*:** Lake Vifilsstadavatn (64°4'47.39"N, 21°52'26.45"W), Iceland.

***Type-specimens*:** Hapantotypes (histological sections and phototypes) were deposited at the Natural History Museum, London, UK, under the accession number NHMUK 2018.235.

***Site in host***: Stomach.

***Prevalence***: 19% (6/31).

***Representative DNA sequences*:** A partial *SSU* rDNA sequence (2070 bp) was deposited in the GenBank database under the accession number MH414927.

***ZooBank registration*:** To comply with the regulations set out in article 8.5 of the amended 2012 version of the *International Code of Zoological Nomenclature* (ICZN) [29], details of the new species have been submitted to ZooBank. The LSID for the new species name *Paramyxidium magi* is: urn:lsid:zoobank.org:act:FEC23203-00E5-4862-8D24-FA5D6D8FFB0C.

***Etymology*:** Specific name derived from Icelandic for stomach ‘magi’

### Description

***Spore.*** Mature spores lemon-shaped in valvular aspect (Fig. 1a), oval to bluntly-rounded in sutural view with sigmoidal sutural line (Fig. 1b), measuring 10.8–12.9 × 7.0–8.4 (11.6 × 7.6) (L × W) (*n* = 20). Polar capsules almost spherical, 3.6–4.6 × 3.1–4.0 (4.0 × 3.6) (*n* = 20), opening sub-terminally (Fig. 1a-b). Fine spore striations visible across both spore valves (Fig. 1c).

### Remarks

Histozoic in the stomach wall forming polysporous plasmodia of variable sizes (*c.*60–95 μm) in the gastric gland interstitium (Fig. 2b). Differs from other *Paramyxidium* spp. primarily on the site of infection and genetic distance.

### *Paramyxidium bulani* n. sp.

***Type-host*:** *Megalops cyprinoides* Broussonet (Elopiformes: Megalopidae).

***Type-locality*:** Kilim mangroves, Langkawi Island, Malaysia.

***Other localities*:** Pangkor Island, Malaysia.

***Type-specimens*:** Hapantotypes (histological sections and phototypes) were deposited at the Natural History Museum, London, UK, under the accession number NHMUK 2018.237.

***Site in host***: Kidney.

***Prevalence***: 29% (4/14).

***Representative DNA sequences*:** A partial *SSU* rDNA sequence (2059 bp) was deposited in the GenBank database under the accession number MH414929.

***ZooBank registration*:** To comply with the regulations set out in article 8.5 of the amended 2012 version of the *International Code of Zoological Nomenclature* (ICZN) [29], details of the new species have been submitted to ZooBank. The LSID for the new species name *Paramyxidium bulani* is: urn:lsid:zoobank.org:act:DA7B51B0-590F-45A5-88CF-32A015B99593.

***Etymology*:** Specific name refers to the local Malay name for the fish host (ikan bulan).

### Description

***Spore.*** Mature spores oval to bluntly-rounded in sutural view, lemon-shaped in valvular aspect, 6.1–6.9 × 4.2–5.1 (6.7 × 4.7) (L × W) (*n* = 20). Polar capsules almost spherical, 2.1–2.5 × 1.5–2.0 (2.3 × 1.8) (*n* = 20), opening sub-terminally. Sutural line sigmoidal and inconspicuous (Fig. 1g-k), spore valves with fine striations.

### Remarks

Plasmodia are elongated and polysporous (size range: length, 25–75 μm; width, 10–22 μm) and develop as intratubular, in both proximal and distal kidney tubules. Differs from other *Paramyxidium* spp. by having much smaller myxospores and infecting the kidney of Pacific tarpon. Stained spores have a sporoplasm containing two nuclei (Figs. 1i and 2d)

### *Paramyxidium branchialis* n. sp.

***Type-host*:** *Anguilla anguilla* (L.) (Anguilliformes: Anguillidae).

***Type-locality*:** Lake Vifilsstadavatn (64°4'47.39"N, 21°52'26.45"W), Iceland.

***Type-specimens*:** Hapantotypes (histological sections and phototypes) were deposited at the Natural History Museum, London, UK, under the accession number NHMUK 2018.236.

***Site in host***: Gills.

***Prevalence***: 83% (25/31).

***Representative DNA sequence*:** A partial *SSU* rDNA sequence (2082 bp) was deposited in the GenBank database under the accession number MH414926.

***ZooBank registration*:** To comply with the regulations set out in article 8.5 of the amended 2012 version of the *International Code of Zoological Nomenclature* (ICZN) [29], details of the new species have been submitted to ZooBank. The LSID for the new species name *Paramyxidium branchialis* is: urn:lsid:zoobank.org:act:2481F7F4-5B9F-42C8-B2EB-FED264FA7283.

***Etymology*:** Specific name refers to site of infection in the host.

### Description

***Spore.*** Mature spores oval to bluntly-rounded in sutural view, lemon-shaped in valvular aspect, 10.7–12.3 × 6.6–7.8 (11.6 × 7.3) (L × W) (*n* = 20). Polar capsules almost spherical, 3.9-4.5 × 3.2-4.2 (4.2 × 3.8) (*n* = 20), opening sub-terminally. Sutural line sigmoidal and inconspicuous. Fine spore striations visible across both spore valves.

### Remarks

Plasmodia are polysporous, differ significantly in size (*c*.35–142 μm), and develop in the gills (Fig. 2c), whereas other species in the genus develop in different host tissues. Differs from other *Paramyxidium* spp. primarily on the site of infection and genetic distance.

## Discussion

Morphological features, such as myxospore size and number of valvular striation, have been used to identify or differentiate between species of *Myxidium* infecting eels in the past [6–10]. However, with such conserved myxospore morphology within the *Paramyxidium* n. g. clade, we do not find this to be a reliable feature to successfully differentiate between species. The location of the parasite within certain specific host tissues would appear to be a far more useful characteristic, with similar tissue tropism being reported for other myxosporean clades in the past [1, 2, 4]. The European eel and its parasites have been extensively studied [5–7, 14] and there have been numerous reports of *Myxidium giardi* infecting the European eel [7, 12, 30]. However, many of these reports of *M. giardi* infections are from tissues other than kidney and it is therefore highly likely that many of these diagnoses are in fact detailing a morphologically similar parasite from the genus *Paramyxidium* but not *M. giardi*. Numerous taxa that fit within the *Paramyxidium* may already be described [8–12], or have been synonymised with other species due to similar spore morphologies [7, 12]. Given the clear differences in *SSU* rDNA data that exist between the known members of this new genus, it is imperative that such data is supplied when validating or describing novel species within this group. The *Paramyxidium* clade currently contains sequences from myxospores that infect various tissues of fish from the superorder Elopomorpha (tarpon/ladyfish and eels). The clade is also comprised of numerous sequences generated from actinospores that probably represent a hidden diversity of unidentified myxosporeans infecting various tissues from anguillid eels; as the sequences have all been generated from European freshwater systems that lack elopiform fish other than eels and *Myxidium*-like infections have been noted in numerous tissues from the European eel [31, 32].

Our phylogenies show that the *Paramyxidium* group is robustly supported as a sister clade to the *Myxidium* clade (*s.s.*) / *Chloromyxum* grouping that form as fully supported subclades (Fig. 3). These three clades all demonstrate levels of fish host association and degrees of tissue tropism. All currently known members of the *Myxidium* clade (*s.s.*) are described from the renal systems of either pike or salmonids (Protacanthopterygii), whilst the adjacent *Chloromyxum* sister group are all described from Cypriniformes, when infecting the gall-bladder (dashed green box, Fig. 3), with the basal species from the clade, *Chloromyxum kurisis* (GenBank: KJ526212), being from the urinary tract of an Atheriniformes fish. This suggests that these two clades have evolved from a common ancestor that infected the renal system of fish potentially having a *Chloromyxum*-like form. The *Paramyxidium*, as a sister to these two groups, have only been described from the Elopomorpha, with the basal species in the clade again coming from a renal infection, further indicating that the common ancestor for the whole grouping was likely to have been a renal myxosporean. A deeper extension of this phenomenon can also be applied to our tree topology. The node from which the Platysporina clade forms as a sister to the *Paramyxidium*/*Myxidium* (*s.s.*) clade, could also represent a common ancestor that infected the renal system of fish. Recently the Myxobilatidae (all renal-infecting) have been shown to be phylogenetically placed within the platysporinids [33], therefore, a renal myxosporean ancestry for the Platysporina is plausible, which could also be associated with a *Chloromyxum*-form. This outcome is not unexpected, as it has been previously demonstrated that the *Chloromyxum* morphotype is important in phylogenetic studies and appears to represent a basal morphotype for numerous myxosporean clades [22, 23, 34]. Therefore, additional sequence data from this basal morphotype, in particular when infecting more ancient fish lineages (Atheriniformes, Cypriniformes, Elopomorpha and Protacanthopterygii), will assist in future phylogenetic studies of the Myxosporea and help to better resolve the relationships between certain reproducible clades.

Certain anomalies do exist in our phylogenetic tree, which can be explained as follows. The *Myxidium* clade (*s.s.*) currently contains myxosporeans that infect the urinary system of fish from the Protacanthopterygii, a superorder of more basal teleosts, which includes the Salmoniformes (salmonids) and Esociformes (pikes). The sequence in the tree representing *Sphaerospora oncorhynchi* Kent, Whitaker & Margolis, 1993 (GenBank: AF201373) is, therefore, more likely to be from a fish that was co-infected with a *Myxidium*, likely related to the *Myxidium* sp. described as the CKX myxosporean from coho salmon in Canada [35]. Indeed, studies have found both *S. oncorhynchi* and *Myxidium salvelini* Konovalov & Shul’man, 1966, in the renal systems from the same *Oncorhynchus* spp. [36, 37] and other striated *Myxidium* spp. have been reported from the urinary systems of salmonids: *Myxidium minteri* Yasutake & Wood, 1957; *M. salvelini* Konovalov et Shul’man, 1966; *Myxidium noblei* Konovalov, 1966; *Myxidium* sp. of Mavor & Strasser, 1918.

The positioning of the sequences *Zschokkella* sp. (GenBank: AJ581918), *Chloromyxum schurovi* (GenBank: AJ581917) and *Myxidium giardi* (GenBank: AJ582213) (dashed black box, Fig. 3), all generated from the same study [13], within a sub-clade of the Myxobilatidae (Platysporina) remains puzzling, especially the *Myxidium*/*Zschokkella* spore forms that exclusively belong to the Variisporina and should not therefore be present in the Platysporina [33]. This concern is reinforced, as the sequence given for *M. giardi* in eels from Scotland is almost identical to that of *C. schurovi* from salmonids [13]. In his description of the genus *Acauda*, Whipps [38] demonstrated the clustering of the genera *Acauda*, *Myxobilatus* and *Hoferellus* in phylogenetic analyses and noted that the myxospores shared similar morphological features, all having longitudinally striated spore valves and a sutural plane that bisects a pair of polar capsules. This combination of features justified the reuse of the family Myxobilatidae Shul’man, 1953, to accommodate these three genera. In addition, it has been demonstrated that *Ortholinea* should be included in the family Myxobilatidae, as *Ortholinea* spp. share similar morphological features, group within the same clade, and collectively they are all parasitic in the renal system of fish [33]. *Chloromyxum* myxospores also share some synapomorphies with this group, having subspherical or elongated spores that may have caudal appendages, with a straight central suture. *Chloromyxum* is known to be polyphyletic genus and the retention of some features (4 polar capsules) in some derived species could explain why evolved chloromyxid forms are present in some clades, such as the Myxobilatidae clade (*C. schurovi*) and the hepatic biliary clade (*Chloromyxum trijugum*). Indeed, Holzer et. al. [39], reported that *C. schurovi*, whilst being genetically similar to members of the clade it occupied (Myxobilatidae), it shares less than 70% identity to other more basal *Chloromyxum* species and concluded it had evolved further than all other sequenced chloromyxids [39]. Therefore, the inclusion of *C. schurovi* with the Myxobilatidae can potentially be explained in terms of DNA sequence, spore morphology and tissue tropism. However, the presence of *Myxidium giardi* (GenBank: AJ582213) and *Zschokkella* sp. (GenBank: AJ581918) still cannot, as spores with these morphologies share no synapomorphies with any members of the clade or the predicted ancestral morphotypes, which are assumed to be chloromyxids. One possibility is that the sequences generated for *M. giardi* and *Zschokkella* sp. either do not correspond to the myxospores that were observed in the tissue samples, or that they were misidentified. They may represent variants of a sequence for *Zschokkella stettinensis* Wierzbicka, 1987, described from the urinary bladder of the European eel [40]. Indeed, the author of the sequences in question also indicated, in their PhD thesis, that *Z. stettinensis* was a possible identity for this myxosporean [41]. The myxospores of *Z. stettinensis* look superficially like those of *M. giardi* but are also very similar to the *Neomyxobolus* morphotype (known to infect the renal system of freshwater fishes), being described as spores having two delicate streaks running parallel to the suture [40], a feature almost unique to *Neomyxobolus* spp. [42]. Therefore, we suggest that these sequences represent variations of *Z. stettinensis* or genetically related forms, which are better placed in the genus *Neomyxobolus* Chen et Hsieh, 1960. *Myxidium streisingeri* Whipps, Murray & Kent, 2015 also groups in the Myxobilatidae and is another species showing several traits in common with the genus *Neomyxobolus* [33], indicating that it has been mistakenly placed as *Myxidium*. Furthermore, these species also have the tissue location (renal system) and spore characteristics to allow placement in the Myxobilatidae.

In the present study, coinfections with other myxosporeans were not observed in Icelandic eels, only *Paramyxidium* morphologies were observed. However, the Pacific tarpon is known to have other myxosporean parasites [4]. Therefore, it is possible when sampling tissues such as the kidney, that blood-borne stages of non-target myxosporean could be amplified in error. This is considered to be unlikely in this case, as most of the myxosporean taxa from the Pacific tarpon from the type location have been identified [4, 43] (MAF, unpublished data). In addition, the fact that these distantly related parasites form a robust monophyletic clade, support the fact that the sequences were derived from the myxospore forms observed.

## Conclusions

*Paramyxidium giardi* n. comb. (syn. *Myxidium giardi*) does not cause systemic infections in eels. In Iceland there are three species now confirmed, using morphological and molecular data. Additional species probably exist that infect different tissues, such as the skin, and a further member of this clade was identified here from an *Aurantiactinomyxon* sequence. Therefore, the site of infection in the host eels is an important diagnostic feature for this group (*Paramyxidium* clade). Myxospore morphology is highly conserved in the *Paramyxidium* clade, and although some myxospore dimensions are noted as significantly different between isolates, differentiating species based on spore dimension alone is not recommended. Despite very similar myxospore morphologies being present across the group there are relatively low genetic similarities between *SSU* rDNA sequences. The *Paramyxidium* clade is a well-supported sister to the *Myxidium* (*s.s.*) and *Chloromyxum* clades, each demonstrating a degree of fish host group specificity.

## Abbreviations

BLAST: Basic Local Alignment Search Tool
LSID: Life Science Identifier
*s.s*: *sensu stricto*
SEM: Scanning electron microscopy
